# Identification of glycosaminoglycan binding regions in the *Plasmodium falciparum *encoded placental sequestration ligand, VAR2CSA

**DOI:** 10.1186/1475-2875-7-104

**Published:** 2008-06-06

**Authors:** Mafalda Resende, Morten A Nielsen, Madeleine Dahlbäck, Sisse B Ditlev, Pernille Andersen, Adam F Sander, Nicaise T Ndam, Thor G Theander, Ali Salanti

**Affiliations:** 1 Centre for Medical Parasitology at Institute of International Health Immunology and Microbiology, University of Copenhagen and Department of Clinical Microbiology, Copenhagen University Hospital (Rigshospitalet), Copenhagen, Denmark; 2 Center for Biological Sequence Analysis, BioCentrum-DTU, Copenhagen, Denmark; 3 Laboratoire de Parasitologie, Institut de Recherche pour le Développement, Université Paris Descartes, Paris, France

## Abstract

**Background:**

Pregnancy malaria is caused by *Plasmodium falciparum*-infected erythrocytes binding the placental receptor chondroitin sulfate A (CSA). This results in accumulation of parasites in the placenta with severe clinical consequences for the mother and her unborn child. Women become resistant to placental malaria as antibodies are acquired which specifically target the surface of infected erythrocytes binding in the placenta. VAR2CSA is most likely the parasite-encoded protein which mediates binding to the placental receptor CSA. Several domains have been shown to bind CSA *in vitro*; and it is apparent that a VAR2CSA-based vaccine cannot accommodate all the CSA binding domains and serovariants. It is thus of high priority to define minimal ligand binding regions throughout the VAR2CSA molecule.

**Methods:**

To define minimal CSA-binding regions/peptides of VAR2CSA, a phage display library based on the entire *var2csa *coding region was constructed. This library was screened on immobilized CSA and cells expressing CSA resulting in a limited number of CSA-binding phages. Antibodies against these peptides were affinity purified and tested for reactivity against CSA-binding infected erythrocytes.

**Results:**

The most frequently identified phages expressed peptides residing in the parts of VAR2CSA previously defined as CSA binding. In addition, most of the binding regions mapped to surface-exposed parts of VAR2CSA. The binding of a DBL2X peptide to CSA was confirmed with a synthetic peptide. Antibodies against a CSA-binding DBL2X peptide reacted with the surface of infected erythrocytes indicating that this epitope is accessible for antibodies on native VAR2CSA on infected erythrocytes.

**Conclusion:**

Short continuous regions of VAR2CSA with affinity for multiple types of CSA were defined. A number of these regions localize to CSA-binding domains and to surface-exposed regions within these domains and a synthetic peptide corresponding to a peptide sequence in DBL2 was shown to bind to CSA and not to CSC. It is likely that some of these epitopes are involved in native parasite CSA adhesion. However, antibodies directed against single epitopes did not inhibit parasite adhesion. This study supports phage display as a technique to identify CSA-binding regions of large proteins such as VAR2CSA.

## Background

PAM (Pregnancy Associated Malaria) is a major health problem in malaria-endemic areas and on a world basis it affects millions of pregnant women and their offspring. The presence of parasites in the placenta of pregnant women can have serious consequences for both mother and child including: maternal anaemia, premature delivery, low birth weight and increased infant mortality [1]. In malaria endemic areas, children acquire clinical immunity after multiple infections, and adults are in general protected against malaria. Women who have acquired immunity against malaria during childhood become susceptible to malaria during pregnancy due to novel parasite phenotypes expressing unique antigens not encountered during childhood infections [2,3]. In areas of high parasite transmission PAM mainly affects primigravidae since immunity is acquired as a function of gravidity [1]. Protective antibodies target proteins expressed on the surface of infected erythrocytes (IE), which mediate binding to syncytiotrophoblasts. By this process, the parasite is not filtered through the spleen and thus avoids exposure to effector mechanisms, which clear erythrocytes infected with late blood stage parasite from circulation [4]. The best characterized surface protein is the *Plasmodium falciparum *erythrocyte membrane protein 1 (PfEMP1) [5,6], which is encoded by the polymorphic *var *gene family containing 50–60 copies per parasite genome [7]. The PfEMP1 family constitutes high-molecular proteins of 200–400 kDa, which are highly polymorphic. Different PfEMP1 molecules have different receptor specificities, therefore switching between expression of various *var *genes in a mutually exclusive manner allows the parasite to modify its adhesion properties (reviewed in [8]). PfEMP1 proteins include three to seven Duffy-binding-like (DBL) domains, which belong to a parasite adhesion-domain super-family present in erythrocyte invasion ligands called: erythrocyte-binding ligands (EBL). Antibodies against PfEMP1 can interfere with parasite binding and the successive acquisition of a broad range of PfEMP1 antibodies is important for the acquisition of immunity during childhood [9-13]. Several molecules such as ICAM-1 [14], VCAM-1 [15], thrombospondin [16], CD36 [17], and chondroitin sulfate A (CSA) [18,19] have been identified as host receptors for PfEMP1. In the placenta IE exclusively bind to the glycosaminoglycan CSA [19,20]. The parasite protein mediating IE adhesion to CSA in the placenta is a distinct member of the PfEMP1 protein family, named VAR2CSA [21]. High levels of anti-VAR2CSA antibodies are correlated with favourable birth outcome and they are acquired as a function of parity [22]. Disruption of the *var2csa *gene causes the loss of the IE's ability to bind CSA [23]. VAR2CSA is a large IE surface-expressed antigen consisting of six DBL domains with a total estimated molecular mass of 350 kDa. The ultimate aim of PAM vaccine development is to define a VAR2CSA construct capable of eliciting antibodies that inhibit binding of IEs to CSA. However, several of the VAR2CSA domains have *in vitro *affinity to CSA [24-26] and this complicates vaccine design. It is thus of high priority to define the minimal epitopes within each domain and inter-domains that have affinity to CSA.

Phage display is a strong and widely used tool for mapping protein ligand interactions and has in several studies been used to define adhesive parts of proteins present on the surface of different organisms causing infectious diseases (reviewed in [27]). Phage display has also been extensively used in malaria research. For vaccine development Casey and others [28], used phage display to isolate a phage-derived peptide that mimic an important epitope of AMA-1 and had the ability to induce functionally protective antibodies. Lanzillotti and others [29], used a phage display library to search for *P. falciparum *encoded motifs involved in erythrocyte invasion, and identified regions in EBA-175 and Ebl-1 like proteins binding to receptors on the human erythrocyte. EBA and Ebl belong to the same super family of Duffy-binding-like proteins as the DBL domains from VAR2CSA. We were thus encouraged to use this technique to search for CSA-binding motifs in VAR2CSA. In this study a phage display library was constructed based on the exon1 coding region of VAR2CSA. The library was biopanned on different sources of glycosaminoglycans (GAG) including: immobilized bovine CSA; immobilized proteoglycans purified from placentas – CSPG; CSA-expressing CHO cells, and BeWo cells derived from the human placental syncytiotrophoblasts. Five regions of VAR2CSA potentially involved in *in vivo *parasite sequestration were identified and are thus potential candidate components of a multivalent PAM vaccine.

## Methods

### Cells

The human choriocarcinoma cell line BeWo was purchased from the American Type Culture Collection [30]. The cells were maintained in Ham's F12 with L-glutamine culture medium (Lonza, Verviers, Belgium) supplemented with 10% FCS and 1% penicilin/streptavidin (Sigma-Aldrich, Ayshire, UK). Chinese Hamster ovary cells (CHO) were maintained in RPMI1640 (with L-glutamine and 25 mM HEPES) (Lonza, Verviers, Belgium) suplemented with 10% FCS and 1% penicilin/streptavidin.

### Construction of the *var2csa *phage display library

The exon1 of *var2csa *was digested with DNase1 (Sigma-Aldrich, Brøndby, Denmark) and fragments of 100 bp–150 bp were ligated into the T7select 415-1b vector (Novagen Inc., Madison, USA) at *EcoRI *and *HindIII *sites. Phage packaging amplification and titration were performed according to the manufacturer's instructions [31].

### Selection of the GAG binding phages from T7*var2csa *library

Different approaches to select for phages binding to CSA were used. Common to these approaches was the general procedure for selection of clones, which consisted in four rounds of biopanning, followed by infection of *E. coli *(BL21) with the selected phages. The bacteria were plated and individual plaques were amplified and sequenced.

(1) Phage selection on human choriocarcinoma cell line BeWo or CHO cell line:

BeWo/CHO cells were cultured in Nuclon™ delta surface (Nunc, Roskilde, Denmark) 6 well-plates until confluent. The plates were incubated for 15 minutes at 4°C before adding 1 × 10^9 ^plaque forming units (pfu) T7*var2csa *phages to the cells for 1 hour at 37°C. The cells were then washed carefully four times in pre-warmed 1% Bovine Serum Albumin (BSA) in PBS for five minutes per wash. The bound phages were eluted in 1 ml of 0.2 M glycin pH 6 for 10 minutes. The eluted phages were amplified to ensure a titer of 10^9 ^pfu for input phages at the start of each successive round.

(2) Phage selection on biotinylated CSA

CSA (C9819 Sigma-Aldrich, Brøndby, Denmark) was biotinylated using EZ-Link Sulfo-NHS-LC-Biotinylation kit (Pierce, Bonn, Germany). 100 μl of 50 μg/ml biotinylated CSA in PBS was added to pre-coated avidin plates (Nunc, Roskilde, Denmark) for 1 hour at room temperature and the wells were subsequently blocked with 200 μl of 1% BSA in PBS for 1 hour. After washing the plates three times with PBS, 1 × 10^9 ^pfu of T7*var2csa *phages were added to the plate for 1 hour at 37°C. The wells were then washed twice in round 1, three times in round 2, and five times in subsequent rounds with TBS 0.5 % Tween-20 for 3 minutes. The phages were eluted in 100 μl of 0.1 M glycin pH 2.2 for 10 minutes, neutralized immediately with 6 μl of 2 M Tris base pH 8 and added to fresh log-phase BL21 cells.

(3) Phage selection on human placenta derived chondroitin sulfate proteoglycans (CSPG) [32] or bovine CSA (Sigma). Proteoglycan (5 μg/ml in PBS) was coated to microtiter plates (Nunc, Roskilde, Denmark) overnight at 4°C. The following steps were done as described for biotinylated CSA.

### PCR amplification of plaques

A portion of top agarose of an individual plaque of interest was scrapped by a pipette tip, dispersed in a tube containing 100 μl of 10 mM EDTA, and heated at 65°C for 10 min. The plaques collected served as templates in a PCR reaction with primers for the T7 vector supplied by the manufacturer (Novagen, Madison, USA). 15 μl of the reaction product was analysed on a 1% agarose gel. For sequencing, the PCR product was purified by Minielute purification kit (Qiagen) and 4 μl of pure PCR product was used in the sequencing reaction.

### Peptide synthesis

Peptides were synthesized by Schafer-N, Århus, Denmark (P2c, P3, P4 and P5) or Sigma (P1, P2a, P2b) (Table 1). Peptides P1, P2a, b, c and P5 were synthesized with the addition of a terminal cystein for increased stability. P2 region is very long and difficult to synthetize and was therefore divided into two peptides P2a and P2c. P2b correspondes to the P2a region and has the FCR3 isolate sequence. PC is a non-VAR2CSA peptide and it was used as a negative control (Table 1). Peptide identity and purity were analysed by MALDI-TOF mass spectrometry and high-performance liquid-chromatography (HPLC). The synthesized peptide sequences are shown in one-letter code (Table 1).

**Table 1:** Amino acid sequence of the synthetic peptides identified by phage display

Peptide ID	Protein ID	Sequence
P1	3D7 – DBL1X	ENQKNKYTELYQQNKC
P2a	3D7 – DBL2X	GKKTQELKNIRTNSELLKEWIIAAFHEGKC
P2b	FCR3 – DBL2X	EDVKDINFDTKEKFLAGCLIVSFHEGKC
P2c	3D7 – DBL2X	LKPSHEKKNDDNGKKLCKAC
P3	3D7 – DBL3X	IKKIIEKGTTKQNGKTVGSGAEN
P4	3D7 – DBL4ε	IKNKNDITNAKKELLETLQIVAERE
P5	3D7 – DBL5ε	ILKGAQSEGKFLGNYYNEDKDKEKALEAMC
PC	*P. falciparum *Potassium pump	WRRDTYNMIWGFNKIPTYIYNMLLILLSTSYID

### Biotinylation of peptides

All peptides were biotinylated using EZ-link Sulfo-NHS-LC-Biotin (Pierce, Bonn, Germany) following the instructions for preferentially biotinylation of N-terminal α-amino groups in peptides [33].

### GAG binding ELISA

**Figure 3:**
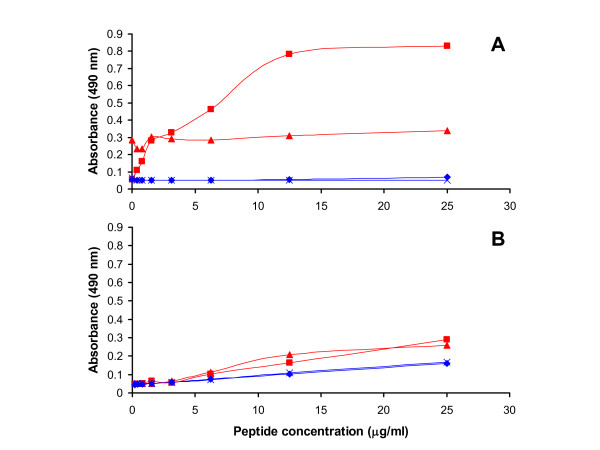
**A DBL2 peptide binds to CSA and not to CSC**. (A) Peptide binding assay to CSA: P2b (red) binds to CSA (■) in a peptide concentration-specific manner and not to the plate (▲). The control peptide (blue) does not bind to neither the plate (×) nor CSA (◆). (B) Peptide binding assay to CSC: P2b (red) does not bind to either CSC (■) or to the plate (▲). The control peptide (blue) does not bind to neither the plate (×) nor CSC (◆).

Flat-bottomed, 96 well plates (Falcon 351172) were coated for 2 hours at 37°C either with CSA (C9819 Sigma-Aldrich, Brøndby, Denmark) 50 μg/ml in PBS; CSC (shark cartilage, Seikagaku, Tokyo, Japan) 50 μg/ml in PBS; or only PBS (as a negative control). The plates were then blocked for 1 hour at 37°C with 1% BSA, 0.05% Tween 20, in PSM buffer (PBS, 2 mM CaCl_2_, 2 mM MgCl_2_, pH 7). After two times washing with 0.05% Tween 20 in PSM buffer, 50 μl of a dilution series (0.4 – 25 μg/ml) of peptide in blocking buffer was added per well and incubated overnight at 4°C. After three times washing, 50 μl of a 1/2000 dilution of a streptavidin-conjugated horseradish peroxidase antibody (Dako, Glostrup, Denmark) in blocking buffer was added to each well and incubated at 37°C for one hour. The binding assay was finalized with three times washing and developing with 100 μl/well of o-phenylenediamine substract for 10 minutes. Plates were read at 490 nm. The curves (Figure 3A) are typical of four independent experiments, and each absorbance value is the mean of two experiments. Figure 3B absorbance values are the result of one experiment.

### Affinity purification of peptide-specific rabbit antibodies

Affinity purification of antibodies was done according tomanufacturer's instructions. In brief, 0,5 mg of peptide was dialyzed against 0.2 M NaHCO_3_, 0.5 M NaCl (pH 8.3), and applied to a NHS-activated HiTrap 1-ml column (GE Healthcare) that had been equilibrated with 3 × 2 ml 1 mM HCl. After coupling, the column was washed with 0.5 M ethanolamine, 0.5 M NaCl (pH 8), 0.1 M acetate, 0.5 M NaCl (pH 4), and a final wash with PBS (pH 7.4). 3 ml of rabbit antisera against the specific domain [34] was subsequently added to the column. After washing in 10 ml PBS, affinity-bound antibodies were eluted by CH_3_COONH_4 _(pH 3) and neutralized in 1 M Tris (pH 7.5). The specificity of the purified antibodies was tested in ELISA against (1) the peptide used for affinity purification, and (2) other VAR2CSA peptides. The affinity-purified antibodies were only positive against the VAR2CSA peptides on which they had been affinity purified.

### Parasite culture and selection

The NF54 laboratory strain of *P. falciparum *was used. Parasite culture was done as previously described [34]. The genotype of the parasite was regularly checked by GLURP and MSP2-specific primers in a single PCR step.

To create a VAR2CSA-expressing parasite population, the NF54 parasites were repeatedly panned on VAR2CSA-specific antibodies creating the NF54^var2csa ^parasite line. Briefly, late stage trophozoites were obtained by gelatin purification and washed twice in RPMI1640. The IE were then incubated for 0.5 hour at 37°C with VAR2CSA-DBL5ε-specific rabbit antiserum and washed three times with medium to remove unbound antibodies. IE expressing VAR2CSA were isolated by the use of protein G-coupled magnetic beads (Dynal, Invitrogen) on a MACS magnet (Miltenyi Biotec). The suspension of IE and beads was washed three times and added to a culture flask with 4% hematocrit of uninfected erythrocytes in 5 ml culture medium. After 24 hours, the beads were removed by magnetic separation. This was repeated until parasites specifically expressed VAR2CSA on the surface. Expression of the VSA_PAM _phenotype was confirmed by specific binding of parasites to CSA in static binding assays, and exclusive recognition of the surface of the IE by antibodies from women exposed to *P. falciparum *during pregnancy.

### Flow cytometry and IFA

Flow cytometry was used to test if the peptide-specific antibodies recognize the surface of CSA-binding IEs. Parasite cultures (NF54^VAR2CSA^) were enriched to contain >75% erythrocytes infected by late trophozoite and schizont stage parasites by exposure to a strong magnetic field [35]. Aliquots (2 × 10^5 ^IE) were labeled by ethidium bromide (to allow exclusion of remaining uninfected erythrocytes). For IE surface staining with rabbit anti-peptide IgG, samples were sequentially exposed to 20 μl sera followed by 1 μl biotinylated sheep-anti-rabbit IgG (The Binding Site, Birmingham, UK) and to 0.5 μl streptavidin-FITC (BD Pharmingen, San Diego, US). All incubations were performed in a total volume of 100 μl PBS with 2% FCS for 30 minutes. Samples were washed two times with 3 ml PBS, 2% FCS between each incubation. Data from a minimum of 5000 IE were acquired using a FC500 flow cytometer (Beckman Coulter, Ramcon, Denmark). For each sample, the mean fluorescence index (MFI) was recorded as a measure of VSA-specific IgG reactivity. Each assay was repeated three times on separate days with similar results. A batch of uninfected erythrocytes was analysed to exclude IgG binding to erythrocyte antigens.

The wet immuno-fluorescent preparations were performed following the staining procedure described for the flow cytometry assays with a few modifications: DNA was labelled with 10 ng/ml DAPI and in the final staining step 0.5 μl Streptavidin-Alexa^®^488 was used instead of FITC (Invitrogen, Paisley, UK). Images were taken using a TE 2000-E Nikon Eclipse confocal microscope, using a 100× Apoplan oil immersion objective. Images were captured using the EZ-C1 Gold imaging system (version 3.30). Images of each parasite preparation stained with VAR2CSA-specific antibodies, peptide-specific antibodies or control antibodies were captured without adjusting pixel dwell time, laser-power nor photomultiplier gain.

## Results

### Multiple linear VAR2CSA regions have affinity for proteoglycans

The exon1 of *var2csa *has 9171 bp and was PCR amplified and cloned into the T7select 415-1b phage vector. The plasmid was propagated and the insert containing the whole *var2csa *exon1 was cut out and digested with DNase1 to generate 200 bp fragments. The *var2csa *fragments were used to create a T7 Phage display library as described in the material and methods section. This vector is described to display 415 copies of peptides on the surface of the T7 capsid [31].

**Figure 1:**
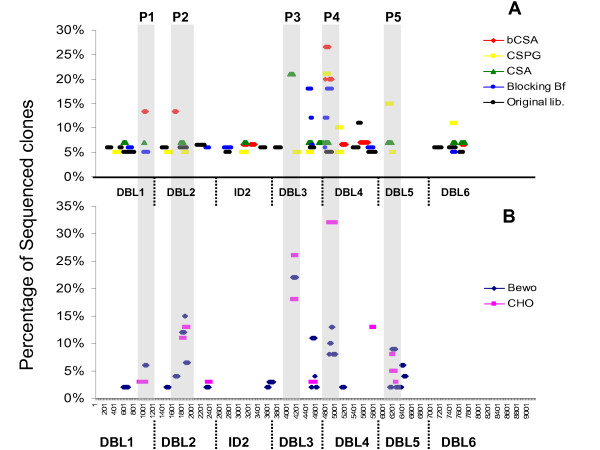
**Frequency of identifed phages sorted according to the identity of the VAR2CSA region and the method of biopanning**. The *var2csa *phage display library was biopanned four rounds on each of the following: biotinylated CSA (A, red); Chondroitin sulfate proteoglycans of human placenta (CSPG) (A, yellow); bovine CSA (A, green); CHO cells expressing CSA on the surface (B, pink) and human placental choriocarcinoma cell line BeWo (B, blue). Control biopannings were done on ELISA plates coated with BSA (A, blue) and the original library was sequenced prior to selection to confirm the presence of sequences belonging to all domains (A, black). For each assay 15 CSA-binding clones were sequenced. Each coloured bar indicates the VAR2CSA sequence expressed by the selected phage on the x-axes and the frequency by which the sequence was detected by sequencing on the y-axes. The length of the coloured bar indicates the length of the VAR2CSA sequence in the selected phage. The VAR2CSA regions most often expressed by the CSA-binding phages are shaded in grey (P1-P5).

To ensure that all parts of VAR2CSA exon1 were present in this constructed library a number of clones were sequenced before biopanning. No sequences were overrepresented and sequences belonging to all six DBL domains were present in the library (Figure 1A, black). The *var2csa *phage display library was biopanned four rounds on: CHO cells (two independent assays); human placental choriocarcinoma cell line BeWo (four independent assays); bovine CSA (two independent assays); biotinylated CSA (two independent assays) and chondroitin sulfate proteoglycans of human placenta (CSPG) (two independent assays). Control biopannings were done on ELISA plates coated with BSA (Figure 1A, Blocking Bf, blue). From each assay 15 clones were sequenced. Figure 1 shows the frequency by which different phages expressing particular VAR2CSA regions were identified after the different types of biopanning. Biopanning on CSA resulted in enrichment of a single region (20% of all phages) corresponding to a peptide sequence in DBL3 (Figure 1A, green). CSA coated directly on ELISA plates might not be very efficient; therefore the biopanning was subsequently repeated using biotinylated CSA (bCSA). This resulted in enrichment of phages representing sequences present in DBL1, DBL2 and DBL4 (Figure 1A, red). In addition, biopanning on human placental CSPG resulted in enrichment of phages representing DBL4 and DBL5 sequences (Figure 1A, yellow). However, the DBL4 sequence was also present in 18% of the control BSA biopannings (Figure 1A, blue). To further identify proteoglycan-binding regions, the VAR2CSA phage display library was biopanned on BeWo and CHO cells expressing CSPG and CSA, respectively (Figure 1B). Enrichment of phages expressing peptides from DBL2, DBL3, DBL4 and DBL5 was seen. Again the DBL4 sequence was also detected in the phages from the control biopanning. DBL2 and DBL3 phages binding to the GAG expressing cells contained VAR2CSA sequences that overlapped with the sequences identified by the CSA biopanning. The DBL5 sequence overlapped with the clone identified by CSPG biopanning. In summary, five relatively short stretches of VAR2CSA appeared to bind soluble GAGs as well as GAGs expressed on cells. No none-DBL regions (i.e. NTS, ID1 or ID2) were identified as GAG binding.

### Mapping the phage display selected regions on models of VAR2CSA DBL domains and comparing with the previous described surface-expressed epitopes

**Figure 2:**
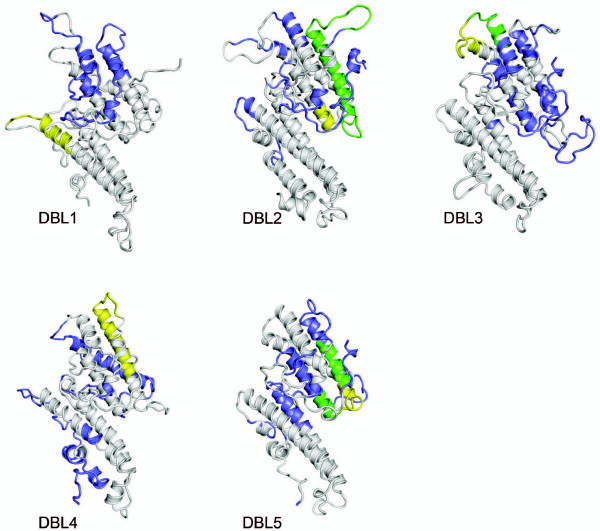
**Structural models of VAR2CSA DBL domains showing surface-exposed regions and GAG binding regions**. The surface-exposed epitopes previously determined by depleting female IgG plasma on parasites expressing VAR2CSA are shown in blue. The GAG binding regions identified by phage display assays are shown in yellow. The overlap of surface-exposed regions and GAG binding regions is shown in green.

Structural models of VAR2CSA 3D7 DBL domains have previously been produced using the solved DBL structures in EBA-175 and Pkα-DBL as templates [36]. As part of previous work we identified regions on native VAR2CSA, which were accessible to antibodies [26,36]. In the current study, the CSA-binding regions defined by the phage display screening were mapped onto the models and compared to the previous findings (Figure 2). Interestingly, in DBL2, DBL3 and DBL5 there was a high degree of overlap between the CSA-binding regions and the surface-exposed regions (Figure 2, green). Peptides residing in DBL1 and DBL4 did not map to the predicted surface exposed regions. All mapped regions, except the DBL1 region, mapped to the S2 subdomain of the DBL domains. The CSA-binding regions mapped in DBL2 and DBL5 are in close vicinity to the chemokine-binding site region of Pkα-DBL Duffy [37].

### Synthetic peptides representing P1-P5

Seven peptides, corresponding to VAR2CSA regions P1-P5 (Figure 1 and Table 1), were synthetized and biotinylated to evaluate their ability to bind CSA in an ELISA assay. For the ELISA based CSA-binding assay: plates were coated with CSA and binding was measured using a streptavidin-conjugated peroxidase-antibody. In order to determine the background binding and detect the true signal, half of the plate was not coated with CSA but all the subsequently steps were performed equally in the whole plate. Using this assay P2b (DBL2) showed specific binding to CSA compared to the background and additionally did not bind to CSC (Figure 3, red curves). A non-VAR2CSA control peptide (Table 1, PC) was used (Figure 3, blue curves) and didn't show binding to any of the GAGs.

### Antibodies specific to the DBL2X peptide react with native VAR2CSA on the surface of infected erythrocytes

**Figure 4:**
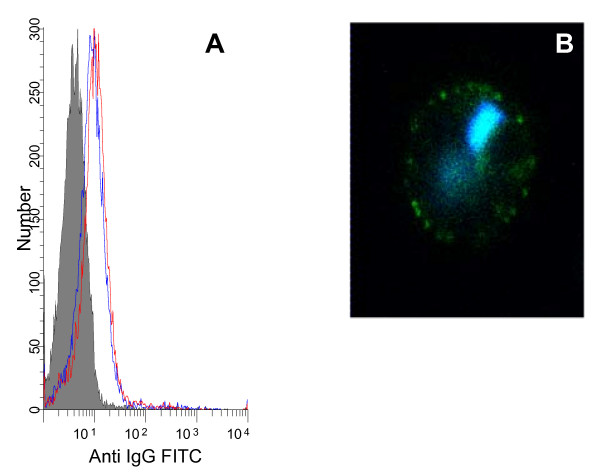
**Anti-P2 antibodies react with native VAR2CSA expressed on the surface of infected erythrocytes**. The histogram (A) shows staining of red blood cells infected with late stages of NF54^var2csa^. The IE reacted with rabbit affinity purified antibodies against P2c peptide (red) and DBL5 (blue). The rabbit prebleed is shown in solid grey. The picture (B) shows an IFA image of IE double stained with anti-P2c antibodies (green) and DNA (DAPI) staining (blue).

Sera from rabbits immunized with recombinant VAR2CSA DBL domains [34] were tested for reactivity against the six peptides. Rabbits immunized with recombinant whole domain DBL1 and DBL4 did not react with the peptides from these domains (P1 and P4). Rabbits immunized with recombinant whole domain DBL2, DBL3 and DBL5 reacted with P2, P3, and P5, respectively. Peptide-specific antibody reagents were then produced by affinity purifying rabbit antibodies on the peptides. The affinity-purified antibodies were tested in flow cytometry for reactivity with native VAR2CSA expressed on erythrocytes infected with the NF54 parasite strain (Figure 4A). Anti-P2c peptide specific antibodies stained the surface of the infected CSA-binding erythrocytes. In addition, the reactivity was observed by wet IFA (Figure 4B). The surface staining determined by IFA showed a dotted pattern typical of PfEMP1 staining. We were unable to detect binding with P2a, P2b, P3 and P5 specific antibodies.

## Discussion

Malaria parasites causing PAM have been shown to bind to glycosaminoglycans in the intevillous space of the placenta. These parasites also bind specifically to bovine CSA [18], CSPG [38] as well as BeWo [39] and CHO cells having CSPG on the surface [18]. The binding between the parasite and the placental CSPG is most likely mediated through the parasite expressed protein, VAR2CSA. VAR2CSA is a large multidomain protein and for vaccine development it is important to define which regions of VAR2CSA are responsible for the interaction with CSPG. In the present study a *var2csa *phage display library was biopanned on five different CSA containing targets (bovine CSA, bovine bCSA, human placental CSPG, BeWo and CHO cells) in 12 independent experiments. Five regions of VAR2CSA repeatedly showed affinity for the different CSA preparations. The CSA-binding peptides identified with the phage display approach were based on the linear sequence of VAR2CSA. However, the CSA binding region might be conformational and involve peptides from several domains. It is preferable that results obtained by phage display assays are confirmed by showing that peptides corresponding to the identified regions also possess binding capacity. The *var2csa *phage display library used in this study was constructed from DNA fragments of 100–150 bp, and the corresponding peptides are thus 34–75 aa. These long peptides were difficult to synthetize and were unstable in solution and we thus had to divide some of the phage regions into several synthetic peptides. Furthermore, the structure of peptides in solution might be very different from peptides bound to a phage. These factors could explain why only one out of seven synthetic peptides could have its binding to CSA confirmed.

DBL2, DBL3 and DBL5 domains of VAR2CSA have previously been shown to bind to CSA [24-26] and the surface-exposed regions within these domains have been mapped [26,36]. Three of the five peptides are located on surface-exposed parts of the previously described CSA-binding domains and two of these peptides map to regions on the DBL domains, which are in close proximity to the ligand-binding region of Pk-alfa-DBL [37]. These findings show an agreement between two independent approaches, which strengthens the present results. No CSA-binding epitopes were found in the highly polymorphic DBL6 domain, which previously has been shown to bind CSA indicating the presence of conformational CSA binding regions in this domain [25].

Region P2 was very long and had to be divided into two peptides for synthesis. The first part of the region showed specific CSA binding (P2b, Figure 3) and the second part of the region (P2c) using peptide affinity purified antibodies in IFA, confirmed that the CS- binding peptide region present in DBL2 was in fact surface-exposed in native VAR2CSA expressed on IE (Figure 4).

The affinity-purified antibodies did not inhibit parasite binding to CSA (data not shown), but this is not surprising as multiple epitopes/domains of VAR2CSA are suggested to be independently involved in CSA binding [24-26]. However, it is possible that all domains of VAR2CSA contribute to a common CSA binding groove, either in a monomeric form or a di-polymeric form, such a glycan binding groove has been shown to be present in a reverse-hand shake dimer of EBA-175 [40]. Individual peptides and domains may have low affinity for binding to CSA, whereas high affinity is only achieved by the assembly of the entire binding site. Chrystallography and structure elucidation of an entire VAR2CSA molecule is not technical feasible with current technology, therefore a complex multi-domain CSA-binding site is difficult to identify. Current methods of identifying the CSA-binding regions of VAR2CSA primarily employ the ability of peptide-specific antibody reagents to inhibit parasite adhesion. If multiple sites are involved in adhesion, screening with single specificity antibodies might not suffice to identify a vaccine construct. By combining the knowledge from: recombinant DBL binding assays; parasite anti-adhesion assays using domain-specific antibodies; epitope mapping approaches and phage display approaches, we might be able to construct a chimeric vaccine, encompassing different CSA binding regions and important antibody epitopes. The phage display approach described here is the first study that attempts to find smaller CSA binding regions within the DBL domains of the vaccine candidate VAR2CSA.

## Conclusion

Phage display was used to identify GAG binding linear regions of VAR2CSA. Five regions located in five different domains were found to have affinity for both immobilized CSA and CSA expressed on the surface of cells. The most frequently observed GAG binding phages mapped to DBL2, 3, 4 and DBL5, and except DBL4 all these domains have been shown to bind CSA *in vitro*. These results are supported by data published by Andersen and others [36], demonstrating that the phage display defined CSA-binding regions in DBL2, 3, 5 all locate to areas of VAR2CSA that appear to be exposed on the native molecule. The DBL2 CSA binding peptide showed specific binding to CSA and affinity-purified antibodies against the same phage display identified region reacted with the surface of infected erythrocytes. This work is the first step in defining small regions of VAR2CSA, which can be used in an adhesion blocking sub-unit vaccine protecting pregnant women against PAM.

## Abbreviations

aa: amino acid; bp: base pair; BSA: bovine serum albumin; CSA: chondroitin sulfate A; DARC: Duffy Antigen Receptor for Chemokines; DBL: Duffy-binding-like; EBA: Erythrocyte Binding Antigen; Ebl: Erythrocyte binding ligand; ELISA: Enzyme-Linked Immunosorbent Assay; FCS: Fetal calf serum; ID1: Inter-Domain region 1 of PfMP1; IFA: Immunofluorescence assay; IgG: immunoglobulin gamma; LBW: low birth weight; NTS: N-terminal segment of PfMP1; PAM: pregnancy-associated malaria; PfEMP1: *Plasmodium falciparum *Erythrocyte Membrane Protein 1.

## Authors' contributions

MR, MAN, MD and SD performed the experiments, PHA plotted GAG binding regions onto the VAR2CSA models, NTN purified the human CSPG used for this study, MR, MAN, NTN, AFS, MD, TGT, and AS were responsible for the study design and the interpretation of data. All authors contributed to writing of the manuscript and approved the final version.
